# Airway pressure release ventilation in acute severe asthma: a case series and physiologic framework

**DOI:** 10.3389/fmed.2026.1901959

**Published:** 2026-08-05

**Authors:** Sajid Kadir, Sumeet Jain, Matthew Kochuba, Firas Madbak, Douglas Kirk, Nicole Clay, Joseph Shiber

**Affiliations:** 1 Division of Pulmonary, Critical Care and Sleep Medicine, Department of Medicine, University of Florida College of Medicine - Jacksonville, Jacksonville, FL, United States; 2 Department of Surgery, University of Florida College of Medicine - Jacksonville, Jacksonville, FL, United States; 3 UF Health Jacksonville, Jacksonville, FL, United States; 4 Department of Emergency Medicine, University of Florida College of Medicine - Jacksonville, Jacksonville, FL, United States

**Keywords:** airway pressure release ventilation, dynamic hyperinflation, extracorporeal membrane oxygenation, mechanical ventilation, respiratory failure, status asthmaticus

## Abstract

**Background:**

Acute severe asthma causes airflow obstruction, dynamic hyperinflation, and progressive hypercapnic respiratory failure. Conventional ventilation prioritizes prolonged expiratory times and permissive hypercapnia to limit air trapping, but some patients develop refractory hypercapnia requiring consideration of extracorporeal support. Airway pressure release ventilation (APRV) is traditionally used in hypoxemic respiratory failure, and its role in obstructive lung disease remains poorly defined.

**Methods:**

We conducted a retrospective case series of adult patients with acute severe asthma who required invasive mechanical ventilation and underwent extracorporeal membrane oxygenation (ECMO) consultation at a tertiary academic center. Clinical characteristics, ventilatory parameters, arterial blood gases, and outcomes were collected. The primary outcome was change in PaCO_2_ and pH following APRV initiation. Secondary outcomes included the need for ECMO, duration of mechanical ventilation, and clinical outcomes.

**Results:**

Six patients with marked hypercapnic respiratory failure (pre-APRV PaCO_2_ 62–136 mm Hg; pH 6.68–7.20) were included. Five received APRV as a rescue strategy; one received APRV as a lung-protective adjunct concurrent with ECMO. Among the five rescue cases, PaCO_2_ decreased to 39–48 mm Hg and pH improved to 7.30–7.41. Time to physiologic improvement (PaCO_2_ ≤ 60 mm Hg and pH ≥ 7.30) ranged from 4 to 48 h. Four of five rescue patients were extubated within 48 h. No new barotrauma or major ventilator-associated complications occurred after APRV initiation.

**Conclusion:**

Following transition to APRV, hypercapnia and acidemia improved and none of the five rescue patients proceeded to ECMO cannulation. Whether this reflects APRV, concurrent medical therapy, or the natural course of treated bronchospasm cannot be determined from this small uncontrolled series. These observations suggest a flow-guided airway pressure release ventilation–time-controlled adaptive ventilation (APRV-TCAV) protocol was feasible and was applied without new barotrauma in refractory obstructive respiratory failure. Prospective controlled evaluation is needed to define APRV’s role and identify patient phenotypes most likely to benefit.

## Introduction

Acute severe asthma requiring invasive mechanical ventilation remains a high-risk clinical scenario characterized by airflow obstruction, dynamic hyperinflation, and elevated intrathoracic pressures (1–4). Conventional ventilatory strategies emphasize low respiratory rates, prolonged expiratory times, and permissive hypercapnia to minimize air trapping and barotrauma (5–7). Despite these approaches, patients with refractory status asthmaticus may develop progressive hypercapnia, hemodynamic compromise, and ventilator-associated complications necessitating consideration of venovenous extracorporeal membrane oxygenation (VV ECMO) (8, 9).

Airway pressure release ventilation (APRV) is a mode of ventilation that maintains sustained airway pressure with intermittent releases to facilitate ventilation while allowing spontaneous breathing and alveolar recruitment (10–12). APRV has been increasingly utilized in acute respiratory distress syndrome (ARDS) (13–15), but its role in obstructive lung disease remains poorly defined. Theoretical concerns include worsening air trapping because of inadequate expiratory time. However, physiologic data suggest that APRV may improve ventilation-perfusion matching, reduce work of breathing, and facilitate carbon dioxide clearance under selected conditions (10, 11, 16).

Given the paucity of clinical data in this population, we present a case series of six patients with acute severe asthma managed with APRV. At our institution, patients with asthma who reach the threshold for ECMO consultation are transitioned to airway pressure release ventilation–time-controlled adaptive ventilation (APRV-TCAV) as the rescue ventilatory strategy. Our aim was to describe the feasibility and safety of this approach, together with clinical characteristics, ventilatory parameters, and outcomes, while exploring the physiologic rationale for APRV in refractory obstructive respiratory failure. This series is descriptive and hypothesis-generating and is not intended to establish efficacy or superiority over conventional ventilation.

## Materials and methods

### Study design and setting

We conducted a retrospective case series at a large tertiary academic medical center, reviewing patients admitted between 2020 and 2025 with life-threatening asthma for whom extracorporeal membrane oxygenation (ECMO) consultation was requested in accordance with institutional practice (8, 9). The study was approved by the Institutional Review Board with a waiver of informed consent given its retrospective design.

### Patient population

We included adult patients aged 18 years or older admitted with a primary diagnosis of acute severe asthma or status asthmaticus who required invasive mechanical ventilation and received an ECMO consultation during their ICU course. Patients were excluded if respiratory failure was attributable to an alternative primary diagnosis (e.g., pneumonia or ARDS), or if they were managed exclusively with non-invasive ventilation.

### Ventilator strategy and APRV protocol

Patients were initially managed using conventional modes with low respiratory rates and prolonged expiratory times in accordance with standard management of severe obstructive lung disease.

In patients with persistent or worsening hypercapnia and acidemia despite conventional ventilation, a transition to airway pressure release ventilation (APRV) was performed as a rescue strategy. At our center, patients with asthma who meet criteria for ECMO consultation are transitioned to APRV-TCAV as the institutional rescue mode rather than undergoing further protocolized escalation of conventional ventilator settings. At our institution, ECMO consultation triggers early multidisciplinary evaluation rather than automatic cannulation, and this pathway applies across etiologies of respiratory failure. The ECMO practitioner assesses hemodynamic stability and the risk of impending arrest. Patients in refractory shock, peri-arrest, or otherwise unable to tolerate the time required for a ventilation trial proceed directly to cannulation, as in Case 1. Patients who are hemodynamically stable, in whom there is time for rescue ventilation to take effect, are transitioned to flow-guided APRV-TCAV while the ECMO team remains actively involved and cannulation remains immediately available should gas exchange, acid-base status, or hemodynamics fail to improve. APRV is therefore not a replacement for ECMO, but a rescue-ventilation step during ECMO evaluation in patients judged clinically stable enough for such a trial. Conventional parameters were therefore not systematically optimized to a predefined target before transition, and the conventional settings reflect the patient’s state at the point of referral rather than an optimized comparator strategy. This series accordingly addresses the feasibility and safety of APRV-TCAV within our ECMO-referral pathway and does not compare APRV against optimized conventional ventilation.

Airway pressure release ventilation was implemented using a standardized institutional protocol (Supplementary Figure 1) with the following principles:

- P_high was set based on the plateau pressure from the preceding conventional ventilator mode to maintain alveolar recruitment while avoiding excessive distending pressure.
- P_low was set at 0 cmH_2_O.
- T_high was set to provide the majority of the ventilatory cycle, allowing sustained airway pressure and facilitating spontaneous breathing.
- T_low was individualized and titrated according to expiratory flow dynamics using the ventilator flow–time waveform. Specifically, T_low was adjusted to terminate expiratory flow at approximately 75% of peak expiratory flow, thereby allowing controlled exhalation while minimizing dynamic hyperinflation and limiting excessive derecruitment. In the early phase of stabilization with periods of marked dynamic hyperinflation, T_low was transiently lengthened to permit greater lung emptying when clinical assessment suggested ongoing air trapping despite the flow-terminated release. As bronchospasm improved, T_low was shortened toward the standard 75% target.
- Because automatic tube compensation was not available on the Servo ventilator, pressure support of 5 cmH_2_O was applied above P_high to offset the resistance of the endotracheal tube and ventilator circuit. Pressure support was applied only to spontaneous breaths above P_high and was not applied during the release phase.

This flow-guided approach to T_low titration was applied iteratively based on serial assessment of ventilator waveforms and gas exchange. The two time settings served distinct functions. T_low was governed by expiratory flow alone, terminating at approximately 75% of peak, and was not adjusted to target minute ventilation or PaCO_2_. T_high, by contrast, was the setting titrated to ventilation: it was set initially within 2.5–6 s and thereafter adjusted by clinical judgment to minute ventilation and gas exchange, shortened to increase minute ventilation when hypercapnia persisted and lengthened as PaCO_2_ fell toward the target of PaCO_2_ ≤ 60 mm Hg and pH ≥ 7.30. In obstructive physiology, T_high was adjusted to balance release frequency and spontaneous ventilation; as PaCO_2_ improved, T_high was lengthened to reduce the frequency of imposed releases, while T_low remained the setting directly governing expiratory emptying. Once this target was met and sustained, weaning proceeded by stepwise reduction of P_high in 2 cmH_2_O decrements, with transition to pressure support once the patient was cooperative and bronchospasm had stabilized.

### Medical management

All patients received concurrent standard-of-care therapy for acute severe asthma throughout their ICU course, including continuous nebulized short-acting beta-2 agonists (albuterol 10 mg/h), inhaled anticholinergics (ipratropium bromide 0.5 mg every 4 h), and systemic corticosteroids (methylprednisolone 40 mg intravenously every 6 h). Ketamine was used as a continuous infusion for bronchodilation (0.1–0.5 mg/kg/h). Intravenous magnesium sulfate (2 g over 20 min) was administered to all patients on arrival. These therapies were continued without modification following transition to APRV and were not altered based on ventilator mode.

Following transition to APRV, neuromuscular blockade, when in use, was discontinued in all patients. Sedation was weaned to the minimum required to maintain patient comfort and cooperation, with the goal of facilitating active spontaneous breathing during the high-pressure phase. Dexmedetomidine was preferred where feasible given its analgesic-sedative properties and favorable effect on respiratory drive. Ketamine, as described above, provided additional sedation.

### Data collection

Electronic medical records were reviewed to extract demographic data and clinical characteristics at the time of ECMO consultation. Baseline asthma characteristics were also abstracted retrospectively, including pre-admission inhaled and oral therapy, documented adherence, leukotriene receptor antagonist use, number of severe exacerbations in the previous year, prior ICU admissions and intubations, available baseline spirometry, smoking or vaping history, blood eosinophil count, total IgE, and prior or current biologic therapy (Supplementary Table 1). Several phenotyping variables, including FeNO, formal atopic status, and nasal polyp status, were not available in the retrospective record and are reported as such.

Ventilator parameters before and after initiation of APRV were recorded, including respiratory rate, tidal volume, airway pressures, and ventilator mode. Arterial blood gas values (pH, PaCO_2_) were collected immediately prior to APRV initiation and at predefined intervals following transition (1 h after initiation and every 4–6 h thereafter).

### Outcomes

The primary outcome was change in PaCO_2_ and arterial pH, following initiation of APRV. Secondary outcomes included the need for ECMO cannulation and duration of mechanical ventilation. Disposition at hospital discharge was also recorded. Changes in sedation requirements and hemodynamic parameters following APRV transition were assessed as additional descriptive outcomes.

## Results

### Patient characteristics

Six patients with acute severe asthma requiring invasive mechanical ventilation were included (Table 1). The median age was 36.5 years (range, 19–57 years), and three patients were female. Presenting triggers varied: two were precipitated by viral illness (Cases 3, 4), one by vaping exposure (Case 6), one by substance use (Case 1), and two had no clear precipitant (Cases 2, 5).

**TABLE 1:** Baseline characteristics of patients with severe asthma.

Case	Age, sex	Comorbidities	Trigger	Pre-APRV pH	Pre-APRV PaCO_2_ (mm Hg)
1	46, F	Asthma	Cocaine, cannabis^†^	6.89	136
2	57, M	Asthma, CHF	Unknown	7.20*	85*
3	46, F	Asthma–COPD overlap‡	COVID-19	7.10	130
4	20, F	Asthma	Upper respiratory infection	6.98	95
5	27, M	Asthma	Unknown	6.68	>125¶
6	19, M	Asthma	Vaping#	7.10	62

APRV, airway pressure release ventilation; CHF, congestive heart failure; COPD, chronic obstructive pulmonary disease; OHCA, out-of-hospital cardiac arrest; F, female; M, male. All patients met institutional criteria for ECMO-team evaluation because of severe hypercapnic acidosis and/or clinical extremis. In hemodynamically stable patients, the consultation threshold was pH < 7.25 with PaCO_2_ ≥ 60 mm Hg despite conventional ventilation; this threshold triggered ECMO evaluation and did not mandate automatic cannulation. *Values obtained from venous blood gas; arterial sampling was not feasible at presentation.

† Toxicology positive for cocaine and cannabis at admission.

‡ Asthma–COPD overlap; primary admission diagnosis was status asthmaticus. ¶Initial PaCO_2_ exceeded the upper measurement range of the analyzer (>125 mm Hg). #Out-of-hospital cardiac arrest with return of spontaneous circulation prior to ICU admission.

All patients presented with severe hypercapnic respiratory failure. Pre-APRV PaCO_2_ ranged from 62 to 136 mm Hg across the cohort, and pH ranged from 6.68 to 7.20, noting that the upper value was obtained from a venous blood gas in Case 2 (Supplementary Figure 2). The most severe degree of hypercapnia was observed in Case 1, with a pre-APRV PaCO_2_ of 136 mm Hg, while the lowest arterial pH was recorded in Case 5 at 6.68, two distinct clinical extremes within the cohort. All patients were considered for ECMO at the time of initial management. Individual clinical and physiologic responses to APRV, including pre- and post-APRV gas exchange, time to improvement, ECMO use, and outcomes, are summarized in Table 2. The narrative below describes the cohort-level patterns, with full individual case descriptions provided in the Supplement. Baseline asthma severity, treatment, and disease burden are summarized in Supplementary Table 1. Adherence was documented as intermittent or absent in all six patients, and baseline phenotyping was limited, with FeNO, atopic status, and nasal polyp status not assessed in any patient. Blood eosinophil counts were below 300 cells/μL in all patients. No patient was receiving biologic therapy at presentation. One patient (Case 1) had been approved for and prescribed omalizumab in 2016 but never initiated it, and outpatient pulmonary follow-up was absent, lapsed, or lost in most patients prior to admission.

**TABLE 2:** Clinical and physiologic response to APRV in severe asthma.

Case	APRV use	Pre-APRV pH	Post-APRV pH	Pre-APRV PaCO_2_	Post-APRV PaCO_2_	Time to improv. (h)	ECMO	Extubation (h from APRV)	Discharge disposition
1	Adjunct to ECMO*	6.89	7.35	136	49	N/A*	Yes	∼77^†^	Home
2	Rescue	7.20^‡^	7.34	85^‡^	47	4	No	46	Home
3	Rescue	7.10	7.41	130	45	7	No	36	Home
4	Rescue	6.98	7.34	95	40	9	No	<24	Home
5	Rescue	6.68	7.32	>125	48	24	No	∼30	Home
6	Rescue	7.10	7.30	62	39	48	No	∼192	Rehabilitation

APRV, airway pressure release ventilation; ECMO, extracorporeal membrane oxygenation; PaCO_2_, arterial partial pressure of carbon dioxide (mm Hg). Time to improvement is defined as the time from APRV initiation to the first arterial blood gas with PaCO_2_ ≤ 60 mm Hg AND pH ≥ 7.30. Extubation timing is reported as hours from APRV initiation. Discharge disposition reflects status at hospital discharge only. Longer-term outcomes (30-day mortality, readmission, functional status) are not reported. *Case 1 received APRV as a lung-protective adjunct concurrent with ECMO; reported post-APRV gas exchange reflects combined APRV and ECMO sweep gas contributions.

† Total duration of invasive mechanical ventilation in Case 1 was approximately 77 h; the patient was decannulated from ECMO on hospital day 3 and extubated the same day.

‡ Pre-APRV values for Case 2 obtained from venous blood gas; arterial sampling was not available at presentation.

### Ventilatory management

Five of 6 patients were transitioned from conventional modes to APRV as a rescue ventilatory strategy. One patient (Case 1) proceeded directly to ECMO for refractory hypercapnia and severe acidosis following cardiac arrest at presentation, with APRV applied concurrently as a lung-protective adjunct rather than a rescue strategy. Refractory hypercapnia and severe acidosis were defined as PaCO_2_ ≥ 60 mm Hg with pH < 7.25 persisting for more than 6 hours despite conventional ventilation, the institutional threshold for ECMO consultation. Case 1 differed from the five rescue cases in that the patient presented in extremis following cardiac arrest, with hemodynamic collapse that did not allow time for a trial of APRV to take effect, so ECMO was initiated immediately and APRV was applied as a lung-protective adjunct. The remaining patients were hemodynamically tolerant of a rescue-ventilation trial, with ECMO held in reserve, before any decision on cannulation. None of the five rescue patients had an absolute contraindication to ECMO. ECMO was not initiated in these patients because each demonstrated sufficient physiologic improvement after APRV initiation to obviate cannulation, with improvement in PaCO_2_, pH, and clinical trajectory during ongoing ECMO-team involvement. The PaCO_2_/pH threshold defined ECMO consultation, whereas cannulation was reserved for patients with refractory gas-exchange failure accompanied by peri-arrest physiology, hemodynamic collapse, clinical deterioration, or failure to improve during monitored rescue ventilation. In Case 1, T_low was set at 1.5 seconds and held constant, meeting the protocol’s 75% peak:end expiratory flow target. Acid-base control was managed through ECMO sweep gas flow, allowing PaCO_2_ and pH to be optimized independent of ventilator mechanics. This approach helped maintain alveolar stability and reduce cyclic stress on the lung while gas exchange was supported extracorporeally. Ventilator settings at three time points, pre-APRV, APRV initiation, and four hours after titration, are shown in Table 3. During APRV, measured peak airway pressure generally reflected P_high when patients were passive. After spontaneous breathing resumed, measured PIP could include the added 5 cmH2O of pressure support applied above P_high. Despite this, APRV peak pressures remained below the recorded pre-APRV peak inspiratory pressures in all cases where conventional-ventilation PIP was available, with a maximum documented APRV PIP of 38 cmH2O across the recorded initiation and 4-h reassessment time points.

**TABLE 3:** Ventilator settings before and after APRV initiation.

	Conventional ventilation							APRV — initiation					APRV — 4 h after titration				
Case	Mode	Vt (mL)	RR (/min)	PIP (cmH_2_O)	MAP (cmH_2_O)	FiO_2_ (%)	PEEP (cmH_2_O)	P_high/ P_low (cmH_2_O)	T_high/ T_low (s)	PIP (cmH_2_O)	MAP (cmH_2_O)	FiO_2_ (%)	P_high/ P_low (cmH_2_O)	T_high/ T_low (s)	PIP (cmH_2_O)	MAP (cmH_2_O)	FiO_2_ (%)
1	SIMV (PRVC+PS)	300	14	55	11	40	5	35/0	10.0/1.5	35	30	35	30/0	10.0/1.5	30	26	35
2	PRVC	440	20	39	14	70	8	35/0	4.0/0.5	35	32	100	30/0	5.0/0.5	35	27	45
3	PCV*	N/A	18	N/A	N/A	80	5	28/0	2.3/1.7	28	17	50	28/0	3.0/1.0	28	21	45
4	PRVC*	400	14	N/A	N/A	50	0	32/0	2.5/2.0	32	14	100	26/0	2.2/1.6	31	22	40
5	PRVC	380	12	48	N/A	40	8	35/0	3.0/1.5	38	26	45	30/0	3.0/1.5	30	20	45
6	SIMV (PRVC+PS)	450	14	52	8	100	5	25/0	5.0/2.0	25	24	40^†^	25/0	5.0/2.0	30	20	60

APRV, airway pressure release ventilation; FiO_2_, fraction of inspired oxygen; MAP, mean airway pressure; PCV, pressure control ventilation; PEEP, positive end-expiratory pressure; PIP, peak inspiratory pressure; PRVC, pressure-regulated volume control; PS, pressure support; RR, respiratory rate; SIMV, synchronized intermittent mandatory ventilation; Vt, tidal volume. Pressures in cmH_2_O. P_low, 0 cmH2O in all cases. Pressure support of 5 cmH2O was applied only to spontaneous breaths above P_high and not during the release phase, due to absence of automatic tube compensation on the Servo ventilator. Across the recorded APRV initiation and 4-h reassessment time points, the maximum documented APRV PIP was 38 cmH2O.

*Settings obtained from outside hospital records; PIP and MAP not available.

†Case 6 FiO_2_ was down-titrated from 100% to 40% at APRV initiation; the patient was a post–out-of-hospital cardiac arrest patient with adequate oxygenation (SpO_2_ > 95%) on lower FiO_2_, permitting de-escalation rather than the protocol’s default 100% carry-over. FiO_2_ was subsequently titrated upward to 60% at the 4-h reassessment based on oxygen saturation.

**FIGURE 1:**
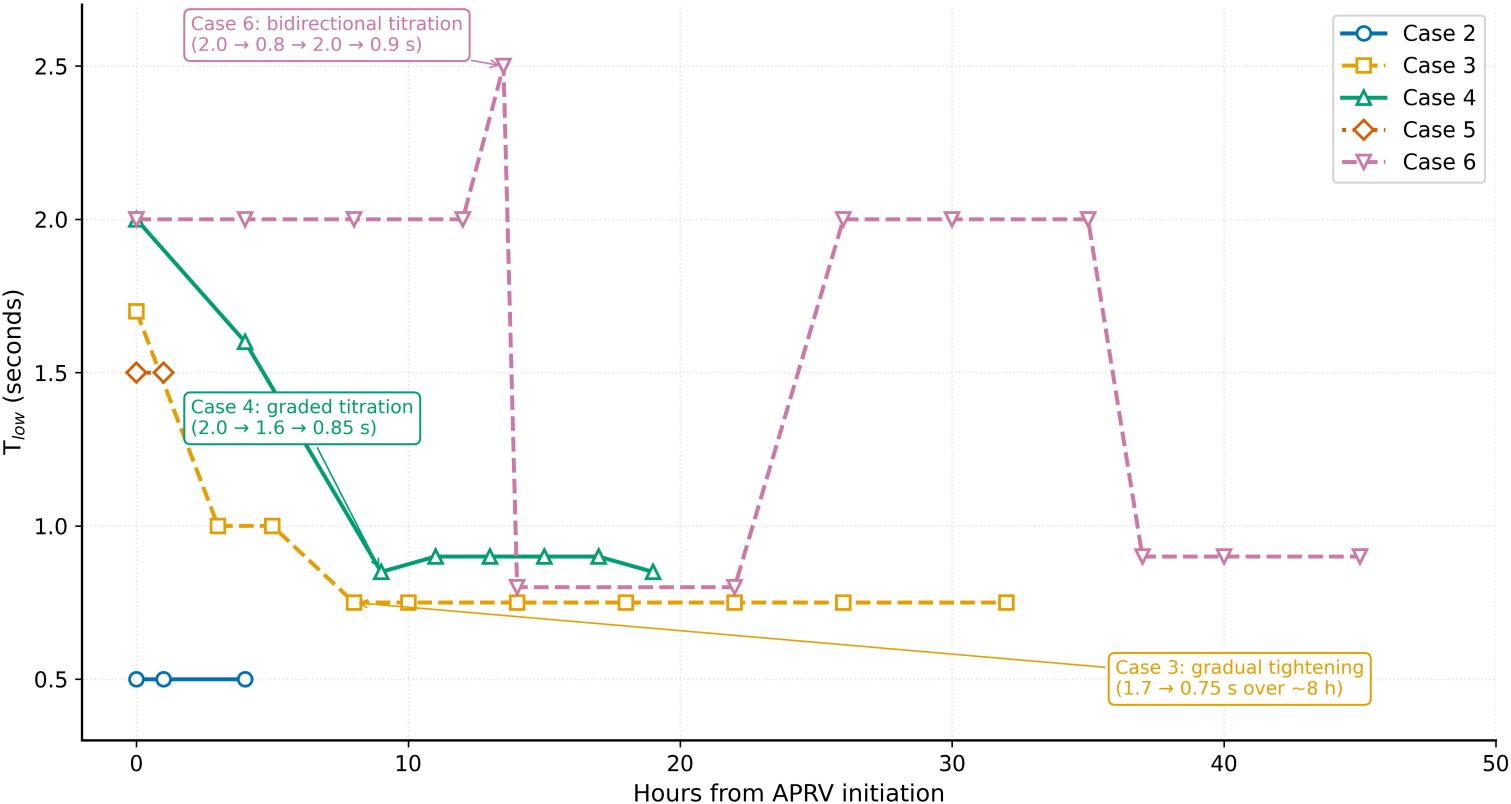
T_low titration trajectories across cases managed with APRV as a rescue strategy. T_low (the brief release phase during which expiratory flow occurs) was individualized at the bedside according to expiratory flow dynamics, with the goal of terminating expiratory flow at approximately 75% of peak. Three patterns emerged: gradual tightening as bronchospasm resolved (Case 3, 1.7 → 0.75 s over ∼8 h); graded titration as airway resistance improved (Case 4, 2.0 → 1.6 → 0.85 s); and bidirectional titration reflecting waxing-and-waning bronchospasm (Case 6, 2.0→2.5→2.0→0.8→2.0→0.9). Cases 2 and 5 had brief APRV courses with limited titration. Case 1 was excluded because APRV was used as an adjunct to ECMO with T_low held constant at 1.5 s. APRV, airway pressure release ventilation; ECMO, extracorporeal membrane oxygenation; T_low, release phase duration. Trajectory shown spans the first 48 h of APRV management; titration continued during subsequent ICU days.

A defining feature of the APRV protocol across the cohort was the individualization of T_low to accommodate obstructive physiology (Figure 1). At the time of APRV initiation, T_low values ranged from 0.5 to 2.0 s, with a mean of 1.53 s. In contrast, in conventional TCAV application in restrictive lung disease, T_low is typically set between 0.2 and 0.5 s to terminate expiratory flow at 75% of peak. The mean T_low of 1.53 s, approximately three times the upper bound of the ARDS range (0.5 s), reflects the prolonged expiratory time constants of severe bronchospasm.

### Gas exchange

Among patients treated with APRV, rapid improvement in ventilation was observed. Among the five rescue cases (Cases 2–6), pre-APRV PaCO_2_ ranged from 62 to 130 mm Hg and decreased to 39–48 mm Hg following APRV initiation, with corresponding improvement in arterial pH to 7.30–7.41 (Supplementary Figures 2A,B). Improvement in ventilation was rapid in most patients. Three of five APRV-treated patients demonstrated improvement (PaCO_2_ ≤ 60 mm Hg and pH ≥ 7.30) within 12 h of initiation: one within 4 h (Case 2) and two within 6–12 h (Cases 3, 4). The remaining two patients showed progressive improvement over 24–48 h; Case 5 met criteria at 24 h and was extubated at approximately 30 h, Case 6 met criteria at 48 h but had a fluctuating course marked by recurrent severe bronchospasm, with gradual improvement and eventual extubation on day 8 after intubation.

### Hemodynamics and sedation

No new hemodynamic instability was observed following APRV transition in any patient. Vasopressor requirements decreased in parallel with improvement in acid-base status and lung mechanics, though hemodynamic data were not systematically collected and this observation should be interpreted with caution. Following transition to APRV, neuromuscular blockade was discontinued and sedation was weaned to a cooperative target in all patients, allowing active spontaneous breathing during the high-pressure phase. No paralytics were used during the APRV period.

### Summary of cases

None of the five patients treated with APRV as a primary rescue strategy proceeded to ECMO cannulation. The single patient who required ECMO did so because of severe acidosis with refractory hypercapnia post-cardiac arrest; APRV was used in this patient as an adjunct to extracorporeal support. Duration of mechanical ventilation was short in most APRV-treated patients, with four of five extubated within 48 hours. One patient required mechanical ventilation for 8 days but was ultimately successfully extubated (Table 2). No new barotrauma was observed following APRV initiation; notably, one patient (Case 5) presented with pneumomediastinum and subcutaneous emphysema that had developed on conventional ventilation prior to APRV transition, and no progression of barotrauma occurred thereafter. All patients survived to hospital discharge; five were discharged home and one was discharged to a rehabilitation facility. Full individual case descriptions are provided in the Supplementary material.

## Discussion

In this series of adults with acute severe asthma and refractory hypercapnic respiratory failure at the threshold of extracorporeal support, transition to a flow-guided APRV-TCAV protocol was feasible and was associated with improvement in gas exchange, no progression to ECMO cannulation in the rescue cases, and short durations of mechanical ventilation, without new barotrauma. The feature that distinguished this approach from prior descriptions of APRV was individualized titration of the release phase to each patient’s expiratory flow dynamics rather than to a fixed release time, anchored to termination of expiratory flow at approximately 75% of peak. Applying this criterion at the prolonged expiratory time constants of severe bronchospasm produced a release phase substantially longer than that used in restrictive disease. Its individualization at the bedside appeared central to ventilating these patients without worsening hyperinflation. These observations were reported across six consecutive patients managed with a single institutional protocol. They establish feasibility but do not, by themselves, demonstrate efficacy of flow-guided APRV in this population. The clinical value of this series lies in describing a reproducible, flow-guided APRV-TCAV approach during ECMO evaluation for acute severe asthma, including patient-selection boundaries, bedside titration principles, and observed short-term safety outcomes in a population for which prior APRV data are limited.

The published experience with APRV is confined almost entirely to acute respiratory distress syndrome and other disorders of normal or increased elastance. The recent expert recommendations on setting and adjusting APRV (17) scope their guidance explicitly to this population and do not address obstructive or decreased-elastance physiology. The supporting clinical literature is similarly situated in ARDS and hypoxemic failure. This literature includes observational rescue cohorts and systematic reviews, as well as a growing body of work favoring early lung-protective application (18–20). To our knowledge, and based on our search, no prior case series has described APRV in acute severe asthma. The few reports in obstructive physiology are isolated cases rather than a protocolized cohort. Our series therefore extends an established flow-guided framework rather than introducing a new one, applying it in a population its originators did not address. It uses the same peak-expiratory-flow termination criterion described in the restrictive setting (21), but at the longer expiratory time constants of bronchospasm (22).

Two features of obstructive physiology distinguish this application from the framework as described for ARDS. First, in the restrictive lung the slope of the expiratory flow waveform is treated as a breath-by-breath surrogate for respiratory system compliance. In acute severe asthma, this slope is dominated by airway resistance rather than by elastance. In this setting, the same waveform-guided titration tracks the resolution of obstruction rather than the recovery of compliance. Second, the transient lengthening of the release phase that we describe during early stabilization uses longer release times than the ARDS setting, where such times are associated with repetitive alveolar collapse. This difference reflects opposing therapeutic goals. In the overdistended, air-trapped lung of status asthmaticus, the aim is a controlled reduction of end-expiratory volume rather than its preservation. In this setting, a longer release serves lung emptying rather than risking derecruitment, although our uncontrolled data cannot confirm this. These considerations also connect to the observation that lengthening expiratory time reduces dynamic hyperinflation in mechanically ventilated severe asthma, though with progressively diminishing returns as expiratory flow falls (23). Our flow-guided approach differs from fixed prolongation in that the release phase is terminated at a defined fraction of the patient’s peak expiratory flow rather than set to an arbitrary duration. Whether this titration yields a measurable reduction in air trapping beyond what expiratory time alone provides cannot be determined from our uncontrolled data. Auto-PEEP was not systematically measured in this series.

### APRV in obstructive lung physiology

Airway pressure release ventilation, as conceptualized within the TCAV framework described by Habashi, is fundamentally a time-controlled pressure strategy rather than one based on tidal volume delivery. Its primary objective is alveolar stability through prolonged periods at elevated pressure, with brief controlled release phases that promote homogeneous gas distribution, reduce cyclic alveolar collapse, and preserve spontaneous breathing (11, 16). The TCAV framework explicitly provides for T_low adjustment based on real-time expiratory flow dynamics, accommodating the prolonged expiratory time constants of obstructive disease. Our protocol applies this framework in the specific context of status asthmaticus at the threshold of extracorporeal support.

**FIGURE 2:**
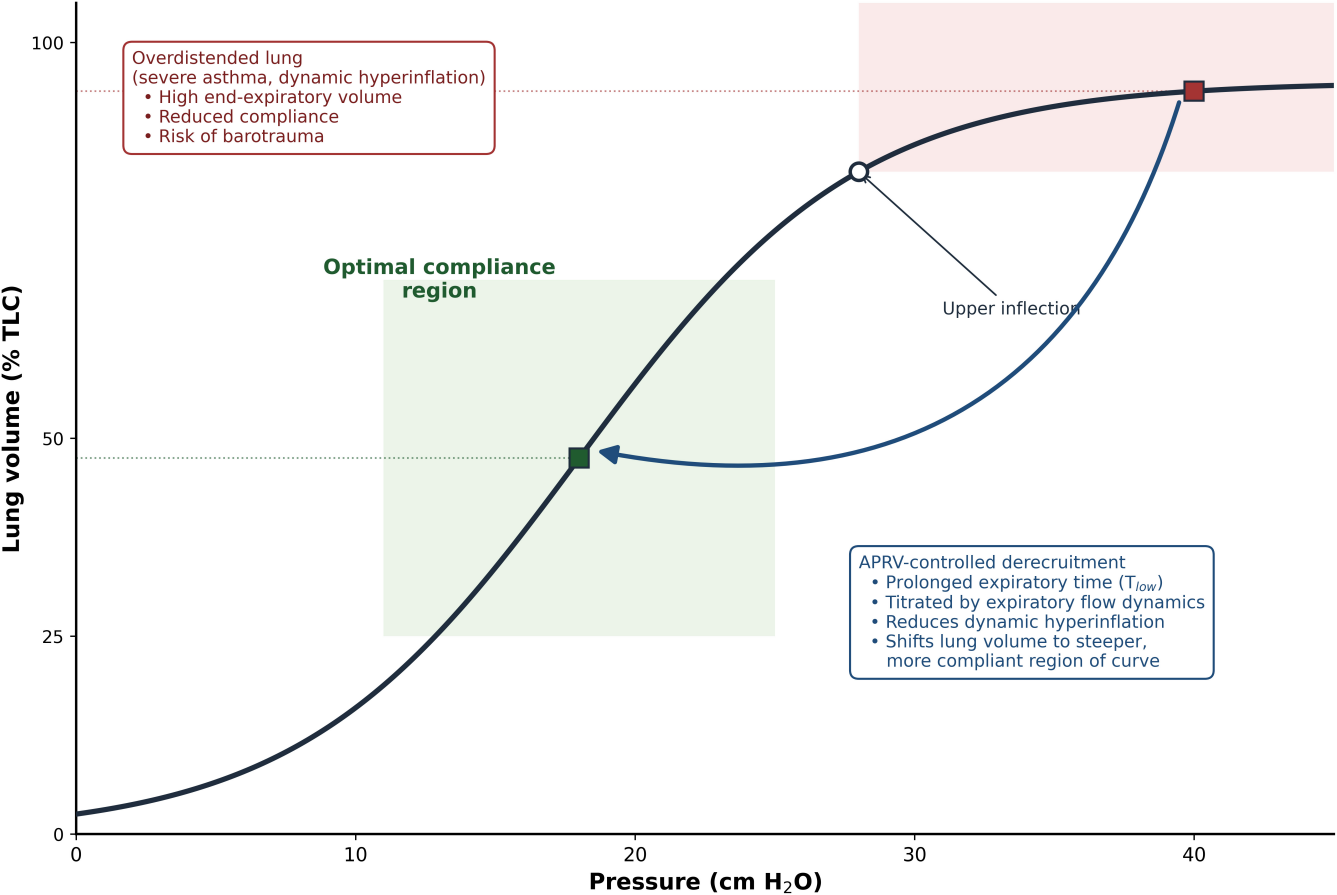
Schematic pressure–volume relationship illustrating the rationale for APRV-guided controlled reduction of end expiratory volume in severe asthma with dynamic hyperinflation. End-expiratory lung volume in patients with severe airflow obstruction sits on the upper, flat portion of the curve (red square), where compliance is reduced and the lung is at risk of barotrauma and impaired venous return. The upper inflection point (open circle) marks the transition from the steep, compliant region to the flat, overdistended region. Prolonged, flow-titrated expiratory time during APRV (T_low set to terminate expiratory flow at 75% of peak) shifts lung volume downward along the curve into the steeper, more compliant region (green square), mitigating dynamic hyperinflation and improving respiratory mechanics. The figure is a conceptual schematic and is not derived from patient data. APRV, airway pressure release ventilation; T_low, release phase duration; TLC, total lung capacity.

**FIGURE 3:**
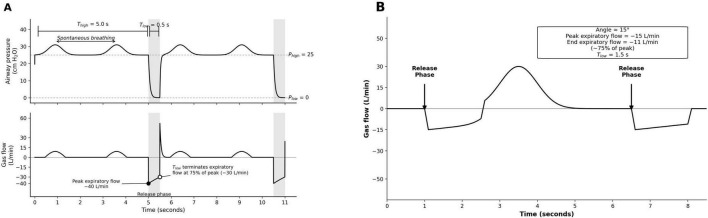
Comparison of expiratory flow waveforms during APRV in normal lung mechanics **(A)** versus severe asthma **(B)**. **(A)** Normal lung mechanics with T_low = 0.5 s. Peak expiratory flow is brisk (–40 L/min) and the release phase angle is steep (∼45°); end-expiratory flow reaches 75% of peak quickly, allowing complete alveolar emptying within a short release phase. **(B)** Severe asthma with T_low = 1.5 s. Peak expiratory flow is markedly reduced (-15 L/min) and the release phase angle is shallow (∼15°), reflecting prolonged expiratory time constants. Termination of expiratory flow at 75% of peak (–11 L/min) is achieved at a release phase approximately three times longer than in normal lung mechanics, reflecting the prolonged expiratory time constants of obstructed lungs. APRV, airway pressure release ventilation; T_low, release phase duration; T_high, high-pressure phase duration; P_high, high pressure setting; P_low, low pressure setting.

In acute reversible obstructive disease, the lung is positioned at or near total lung capacity due to dynamic hyperinflation and incomplete alveolar emptying (24, 25). Unlike ARDS, the therapeutic goal here is different. We focus not on recruitment of collapsed alveoli, but on controlled lung emptying, fundamentally controlled decruitment, reducing end-expiratory lung volume from the overdistended upper limb of the pressure–volume curve toward a more compliant region (Figure 2). To accomplish this, T_low must initially be long enough to allow expiratory volume to exceed inspiratory volume with each breath, leading to net exhalation and gradual lung emptying. The difference between normal (T_low 0.5 s) and obstructed (T_low 1.5 s) release phases is shown in Figures 3A, B.

Clinically, management of severe asthma requires longer T_low values than in ARDS (0.2–0.5 s), consistent with the mean T_low of 1.53 s in this cohort. Peak expiratory flow rate is characteristically low (−10 to −15 L/min), producing a shallow, obtuse angle on the expiratory flow-time waveform. As bronchospasm resolves, peak expiratory flow accelerates toward −40 to −50 L/min, the release phase angle sharpens toward 45 degrees, and T_low can be incrementally shortened, as demonstrated in Cases 3 and 4, approaching the 0.75–0.85 s range, closer to the time constant of normal lung mechanics. In contrast, Case 6 required bidirectional titration, reflecting the waxing-and-waning nature of recurrent bronchospasm. This progressive emptying also augments CO_2_ clearance as compliance improves. In our cohort, T_low was individualized at the bedside to each patient’s expiratory flow dynamics, which we believe was central to safely ventilating these patients without worsening hyperinflation.

In addition to T_low manipulation, sustained pressure during P_high may stent open distal airways, reduce regional airway closure, and improve distribution of ventilation to poorly ventilated lung units. Spontaneous breathing during APRV has been shown to favor ventilation of dependent lung regions in models of acute lung injury (26–28); whether this translates to obstructive physiology is unproven but mechanistically plausible. APRV’s emphasis on time at pressure rather than cyclic volume delivery may also mitigate ventilator-induced lung injury by reducing the repetitive tidal stress imposed on an already mechanically disadvantaged airway (3, 11, 14, 29).

This approach differs importantly from APRV in COPD exacerbation. In chronic obstructive disease, expiratory flow limitation is persistent rather than acutely reversible, and T_low is set longer than in normal lungs typically 0.6–0.8 s. It does not undergo the dynamic shortening that characterizes successful asthma management. COPD decompensation most often reflects a superimposed process, commonly infection, acting on a fixed, baseline prolonged time constant. The TCAV strategy of terminating expiration at 75% of peak expiratory flow remains appropriate to maintain lung stability without overdistension or derecruitment. The clinically important distinction is this: in asthma, T_low is a moving target that should shorten as the patient improves; in COPD, it is a stable parameter matched to a fixed physiologic state.

While our series supports a physiologic role for APRV in severe asthma, the approach carries specific risks that deserve recognition. The most consequential is inadequate expiratory time. If T_low is set too brief, as is appropriate in restrictive lung disease, progressive air trapping and worsening auto-PEEP may result, which will contribute to dynamic hyperinflation. The elevated mean airway pressures inherent to APRV may increase intrathoracic pressure, impair venous return, and worsen right ventricular afterload, a very relevant concern in patients who are already hemodynamically compromised by the effects of severe auto-PEEP. APRV is a pressure-targeted mode; tidal volumes during release phases are determined by the prevailing respiratory system compliance and resistance rather than by a fixed set parameter. Of note, in asthma, with high airway resistance, this may result in unpredictable tidal delivery that requires close monitoring. These risks do not preclude the use of APRV in asthma but underscore the importance of individualized T_low titration, careful hemodynamic monitoring, and selection of patients in whom the potential benefit of achieving physiologic improvement before cannulation outweighs the technical demands of flow-guided pressure ventilation.

The ethical concern in this setting is whether a rescue ventilatory strategy could delay or replace indicated ECMO. In our pathway, this risk is mitigated by early ECMO consultation, continuous multidisciplinary reassessment, and immediate availability of cannulation if the patient deteriorates or fails to improve. APRV was not used to avoid ECMO in patients who required emergent extracorporeal support; rather, it was applied as a monitored rescue-ventilation trial in patients who were clinically stable enough to permit reassessment. The decision not to cannulate the five rescue patients reflected their subsequent physiologic improvement, not the presence of ECMO contraindications. Accordingly, these observations should not be interpreted as evidence that APRV is superior to ECMO or as a recommendation to defer ECMO when clinically indicated.

### Limitations

This case series has several limitations. As a retrospective single-center study of six patients without a control group, causality cannot be established, and the observed improvements in ventilation cannot be clearly attributed to APRV. Generalizability is further limited by our inclusion criterion of ECMO consultation, which introduces both severity and referral bias. Our cohort represents only patients whose clinical course was severe enough to prompt extracorporeal support evaluation, while those who improved with conventional ventilation before reaching that threshold were excluded. These findings should therefore not be extrapolated to the broader population of mechanically ventilated patients with severe asthma. Importantly, conventional ventilator settings were not systematically optimized to a protocolized target before transition, because APRV-TCAV is the institutional rescue mode at the point of ECMO referral. This series therefore cannot address whether APRV is superior to optimized conventional ventilation; it speaks only to the feasibility and safety of APRV-TCAV within our referral pathway. The favorable course observed may reflect APRV, concurrent medical therapy, the natural history of treated bronchospasm, or a combination of these, and these possibilities cannot be distinguished in an uncontrolled design.

Several factors also limit attribution of the observed improvement specifically to APRV. All patients received concurrent standard therapies, including continuous bronchodilators, systemic corticosteroids, ketamine, and intravenous magnesium, each of which may have contributed to the improvement in gas exchange. The independent effect of APRV cannot be separated from these interventions in a retrospective study. In addition, although dynamic hyperinflation and auto-PEEP are central to the physiologic rationale presented here, auto-PEEP data were not consistently available and were not systematically collected. As a result, the proposed reduction in dynamic hyperinflation cannot be directly confirmed. For the same reason, decisions to transiently lengthen T_low were guided by bedside clinical assessment rather than by direct measurement of lung mechanics. Persistent overdistension and incomplete lung emptying were therefore inferred rather than quantified, and we cannot exclude that factors other than air trapping contributed to the waveform and gas-exchange changes that prompted these adjustments. Persistent air trapping or overdistension was inferred from an integrated bedside assessment, including prolonged expiratory flow deceleration, low peak expiratory flow, persistent hypercapnia or acidemia despite the flow-terminated release and clinical assessment of obstructive physiology. Because auto-PEEP, end-expiratory lung volume, and formal respiratory mechanics were not systematically measured, this inference could not be objectively confirmed.

Two practical considerations also deserve emphasis. As described in the Results, Case 1 received APRV as an adjunct to ECMO rather than as a primary rescue strategy and should be interpreted separately. More broadly, the T_low titration strategy central to our protocol is operator dependent. Terminating expiratory flow at 75% of peak flow requires real-time interpretation of the ventilator flow–time waveform at the bedside, and this may vary between clinicians. The generalizability of this approach therefore depends on familiarity with flow-guided APRV titration, which may not be universal.

The baseline characterization of the cohort also has limitations. Acute severe asthma is heterogeneous, and clinical course may differ across undertreated patients, frequent exacerbators, those with asthma–COPD overlap, and those already receiving high-intensity therapy. Although we abstracted available data on severity, treatment, adherence, and disease burden (Supplementary Table 1), several phenotyping variables, including FeNO, atopic status, nasal polyp status, and total IgE, were not systematically available in the retrospective record. The cohort was notable for limited maintenance therapy and intermittent or documented non-adherence in all patients, and for the near-absence of biologic therapy despite features of severe disease. No patient was receiving a biologic at presentation, one had been approved for omalizumab but never initiated it, and pulmonary follow-up was frequently lapsed. Notably, blood eosinophil counts were below 300 cells/μL in all patients, and undertreatment and non-adherence were the dominant baseline features of the cohort. These observations nonetheless align with an emerging literature on type 2-targeted biologics in acute and severe exacerbations (30–32), and suggest that systematic severe-asthma phenotyping and consideration of biologic eligibility, both during and after critical illness, may be relevant to this population, though our small uncontrolled series cannot address this directly.

## Conclusion

Acute severe asthma refractory to conventional mechanical ventilation represents one of the most challenging scenarios in critical care. In this case series, APRV was associated with rapid improvement in hypercapnia and arterial pH in patients with severe status asthmaticus, and none of the five rescue patients proceeded to ECMO cannulation. APRV-TCAV was thus successfully applied in a small series of patients with severe status asthmaticus and was associated with favorable clinical outcomes. Given the retrospective design, small sample, absence of a comparator group, and incomplete physiologic data, these observations are hypothesis-generating and do not establish efficacy or a specific benefit of APRV over conventional ventilation.

The key technical determinant of safe APRV application in this population appears to be individualized, flow-guided titration of T_low using real-time expiratory flow dynamics. Clinicians can tailor alveolar emptying, minimize dynamic hyperinflation, and preserve the ventilatory and hemodynamic benefits of sustained airway pressure by targeting termination of expiratory flow at 75% of peak expiratory flow. This flow-guided approach is central to the protocol described here and distinguishes it from prior unstructured applications of APRV in obstructive physiology.

These findings should be considered hypothesis-generating. Prospective studies comparing flow-guided APRV to conventional lung-protective ventilation in severe asthma are needed. Such studies should incorporate standardized T_low titration protocols to address the limitations inherent to retrospective case series. We recommend that APRV in acute severe asthma be reserved for carefully selected patients with refractory hypercapnic failure and applied by practitioners familiar with flow-guided ventilator management. It should be integrated within a multidisciplinary framework that includes early ECMO consultation as a parallel safety net.

## Data Availability

The de-identified data supporting the findings of this study are available from the corresponding author upon reasonable request, subject to institutional data-sharing policies and approval from the University of Florida Institutional Review Board. Individual patient-level data are not publicly available due to the small cohort size and the consequent risk of re-identification.

## Supplementary material

Supplementary Figure 1 APRV-TCAV protocol. Standardized protocol for transition from conventional mechanical ventilation to airway pressure release ventilation (APRV) in patients with severe asthma and refractory hypercapnia. The protocol comprises six sequential stages: (1) patient selection criteria, requiring pH < 7.25 with PaCO_2_ ≥ 60 mm Hg for >6 h on conventional ventilation and ECMO consultation triggered or active; (2) pre-conversion assessment to record plateau pressure, peak expiratory flow, hemodynamic status, and concurrent therapies; (3) initial APRV settings, with P_high anchored to the prior plateau pressure (typically 25–35 cmH_2_O), P_low set at 0 cmH_2_O, T_high of 2.5–6 s initially with adjustment based on minute ventilation and gas exchange, and T_low of 0.5–2.0 s as a starting point; (4) flow-guided titration of T_low, the critical step, with the goal of terminating expiratory flow at 75% of peak expiratory flow; (5) response assessment based on PaCO_2_ trajectory and pH, with the improvement target defined as PaCO_2_ ≤ 60 mm Hg and pH ≥ 7.30 (or a clear trajectory toward these values), continuing APRV if improving and escalating to venovenous extracorporeal membrane oxygenation (VV ECMO) if not; (6) weaning, with stepwise reductions in P_high in 2 cmH_2_O decrements, adjustment of T_high based on minute ventilation, PaCO_2_, and pH, while T_low remained governed by expiratory-flow dynamics targeting termination at approximately 75% of peak expiratory flow, and transition to pressure support when the patient is cooperative and bronchospasm has stabilized. ABG, arterial blood gas; APRV, airway pressure release ventilation; ECMO, extracorporeal membrane oxygenation; FiO_2_, fraction of inspired oxygen; MAP, mean airway pressure; NMB, neuromuscular blockade; PaCO_2_, arterial partial pressure of carbon dioxide; PaO_2_, arterial partial pressure of oxygen; PEFR, peak expiratory flow rate; TCAV, time-controlled adaptive ventilation; VV ECMO, venovenous extracorporeal membrane oxygenation.

Supplementary Figure 2 Improvement in arterial pH **(A)** and PaCO_2_ **(B)** following APRV initiation in five patients managed with APRV as a rescue strategy (Cases 2–6). Reference lines mark the lower limit of normal pH (7.35) and upper limit of normal PaCO_2_ (45 mm Hg). Time from APRV initiation to the first arterial blood gas meeting the improvement criterion (PaCO_2_ ≤ 60 mm Hg AND pH ≥ 7.30) ranged from 4 to 48 h and is shown for each case in Table 2. Case 1 was excluded because gas exchange during the APRV period was managed by ECMO sweep gas flow rather than ventilator mechanics. APRV, airway pressure release ventilation; ECMO, extracorporeal membrane oxygenation.
