# Supplementary material for: Airway pressure release ventilation in acute severe asthma: a case series and physiologic framework
Source: Front Med (Lausanne). 2026 Aug 5;13:1901959. doi: 10.3389/fmed.2026.1901959 (PMC13485887; doi:10.3389/fmed.2026.1901959)
Supplement: Supplementary file 1 [file Data_Sheet_1.docx]

**SUPPLEMENTARY MATERIAL**

*Airway Pressure Release Ventilation in Acute Severe Asthma: A Case Series and Physiologic Framework*

**Individual Case Descriptions**

The following narratives describe each of the six patients included in the case series. Cohort-level findings and physiologic patterns are presented in the main manuscript; these narratives provide additional clinical context for individual cases. Our APRV-TCAV protocol reflects APRV use as a rescue strategy before ECMO escalation and applies primarily to Cases 2–6. Case 1 received APRV concurrently with VV ECMO as a lung-protective adjunct.

**Standard medical therapy.** All patients received standardized severe-asthma medical therapy throughout their ICU course, including intravenous methylprednisolone, continuous nebulized albuterol, ipratropium, intravenous magnesium sulfate, and ketamine. Sedation was managed with dexmedetomidine and ketamine in all cases. Neuromuscular blockade was used during the initial period of conventional ventilation; paralytic infusions were discontinued at APRV initiation in Cases 2, 3, 4, and 5. Cases 1 and 6 did not receive paralytic infusions at any point. Daily chest radiographs were obtained in all patients; no new barotrauma was identified on serial imaging in any case after APRV initiation.

**Case 1**

*46-year-old woman · status asthmaticus · in-hospital cardiac arrest · ECMO + APRV adjunct · survived to discharge.*

A 46-year-old woman with current tobacco use, asthma and no prior intubations presented with acute respiratory failure following recent cannabis and cocaine use. She developed progressive hypercapnia despite noninvasive ventilation and experienced in-hospital cardiac arrest with return of spontaneous circulation (ROSC) within 10 minutes. Arterial blood gas analysis post ROSC demonstrated severe respiratory acidosis with a nadir pH of 6.89 and PaCO₂ of 136 mm Hg. Venovenous ECMO was initiated emergently for refractory hypercapnia with severe acidosis. APRV was applied concurrently as a lung-protective adjunct to ECMO support, with T_High set at 10 seconds and T_low set at 1.5 seconds. Acid–base control was managed through ECMO sweep gas flow, allowing PaCO₂ and pH to be optimized independent of ventilator mechanics. The patient improved rapidly, was decannulated from ECMO on hospital day 3, extubated the same day, and discharged home.

**Case 2**

*57-year-old man · asthma and CHF · hypercapnic failure · APRV rescue · extubated hospital day 2 · home.*

A 57-year-old man with asthma and congestive heart failure (heart failure with preserved ejection fraction) presented with acute hypercapnic respiratory failure requiring intubation after failed noninvasive ventilation. Initial venous blood gas at presentation revealed pH 7.20 and PvCO₂ 85 mm Hg; arterial sampling was not acquired at presentation. APRV was initiated as a rescue strategy, resulting in improvement of pH and reduction in PaCO₂ within hours. The patient was transitioned to pressure support ventilation the following day and extubated on hospital day 2. ECMO was avoided, and he was discharged home without complications.

**Case 3**

*46-year-old woman · asthma–COPD overlap · COVID-19 trigger · APRV rescue · extubated 36 hours · home.*

A 46-year-old woman with asthma–COPD overlap presented with respiratory failure following COVID-19 infection. She required intubation for respiratory arrest. The patient was initially managed at an outside hospital and transferred for higher-level care, with values of PaCO₂ ~130 mm Hg and pH ~7.10 at the time of transfer. APRV was initiated, leading to rapid improvement in ventilation (PaCO₂ ~130 → 45 mm Hg) and normalization of pH. She was extubated at 36 hours of APRV initiation and discharged home after a brief hospital course without need for ECMO.

**Case 4**

*20-year-old woman · asthma · viral URI trigger · APRV rescue · extubated <24 hours · home.*

A 20-year-old woman with asthma managed on rescue inhaler therapy alone, with no prior intubations, presented with rapidly progressive respiratory failure following an upper respiratory infection. Initial arterial blood gas demonstrated pH 6.98 and PaCO₂ 95 mm Hg with significant dynamic hyperinflation and elevated airway pressures on conventional ventilation. Following initiation of APRV, ventilation improved rapidly with normalization of pH and PaCO₂ within 24 hours. She was extubated 22 hours after APRV initiation and discharged home shortly thereafter without need for ECMO.

**Case 5**

*27-year-old man · status asthmaticus · pneumomediastinum on conventional ventilation · APRV rescue · extubated ~30 hours · home.*

A 27-year-old man with a history of poorly controlled asthma, presented with profound hypercapnic respiratory failure (pH 6.68; initial PaCO₂ exceeded the upper measurement range of the analyzer, reported as >125 mm Hg). Imaging at presentation, demonstrated pneumomediastinum and subcutaneous emphysema, consistent with barotrauma sustained on conventional ventilation prior to APRV initiation. APRV was subsequently initiated with focus on reducing airway pressures and improving expiratory flow dynamics. At the 6-hour mark, PaCO₂ had declined from >125 to 71 mm Hg with corresponding pH correction (6.68 → 7.20). With further time and resolution of bronchospasm, his arterial blood gas had normalized (pH 7.32, PaCO₂ 48 mm Hg) by approximately 24 hours, and the patient was extubated approximately 30 hours after APRV initiation. ECMO was avoided. He was discharged home subsequently.

**Case 6**

*19-year-old man · vaping-associated bronchospasm · out-of-hospital cardiac arrest · APRV with bidirectional T_low titration · extubated hospital day 8 · rehabilitation.*

A 19-year-old man with a history of asthma and vaping presented after out-of-hospital cardiac arrest due to severe bronchospasm associated with vaping exposure; return of spontaneous circulation was achieved prior to ICU admission. His down time was less than 10 minutes. Initial arterial blood gas demonstrated pH 7.10 and PaCO₂ 62 mm Hg. During his ICU course, APRV was utilized to manage recurrent episodes of severe bronchospasm and dynamic hyperinflation and required bidirectional T_low titration (2.0 →2.5 →2.0→0.8 → 2.0 → 0.9 seconds) reflecting the waxing-and-waning nature of his airflow obstruction. Gas exchange first met the predefined improvement criterion at 48 hours but recurrent waxing-and-waning bronchospasm prevented extubation until hospital day 8. The patient was extubated on hospital day 8 and discharged to a rehabilitation facility with subsequent recovery.

Abbreviations: ABG = arterial blood gas; APRV = airway pressure release ventilation; ECMO = extracorporeal membrane oxygenation; FiO₂ = fraction of inspired oxygen; MAP = mean airway pressure; NMB = neuromuscular blockade; PaCO₂ = arterial partial pressure of carbon dioxide; PaO₂ = arterial partial pressure of oxygen; PEFR = peak expiratory flow rate; TCAV = time-controlled adaptive ventilation; VV ECMO = venovenous extracorporeal membrane oxygenation.
