# Supplementary material for: Airway pressure release ventilation in acute severe asthma: a case series and physiologic framework
Source: Front Med (Lausanne). 2026 Aug 5;13:1901959. doi: 10.3389/fmed.2026.1901959 (PMC13485887; doi:10.3389/fmed.2026.1901959)
Supplement: Supplementary file 2 [file Table_1.docx]

**Supplementary Table 1. Baseline asthma severity, phenotype, treatment, and disease burden.**

| **Variable** | **Case 1** | **Case 2** | **Case 3** | **Case 4** | **Case 5** | **Case 6** |
| --- | --- | --- | --- | --- | --- | --- |
| **Age, years** | 46 | 57 | 46 | 20 | 27 | 19 |
| **Sex** | Female | Male | Female | Female | Male | Male |
| **Asthma phenotype / label** | Severe asthma | Severe asthma | Asthma–COPD overlap | Asthma | Asthma | Asthma |
| **Pre-admission rescue therapy** | SABA (albuterol MDI + nebulized) | SABA (albuterol MDI) | SABA (albuterol MDI + nebulized) | SABA (albuterol MDI) | SABA (albuterol MDI + nebulized) | SABA (albuterol MDI) |
| **Maintenance ICS/LABA or triple therapy** | ICS/LABA (fluticasone-salmeterol + budesonide-formoterol) | ICS/LABA (budesonide-formoterol) | ICS/LABA/LAMA triple (fluticasone-umeclidinium-vilanterol) | None (rescue inhaler alone) | ICS/LABA (fluticasone-salmeterol) | ICS/LABA (fluticasone-salmeterol) |
| **Leukotriene receptor antagonist** | Montelukast | None | Montelukast | None | Montelukast | Montelukast (d/c 2019) |
| **Maintenance / PRN oral corticosteroid** | PRN prednisone burst | None | PRN prednisone burst (ED) | PRN prednisone taper (ED) | PRN prednisone taper | None |
| **Adherence** | Intermittent (financial/insurance) | Documented non-adherence | Intermittent (financial/insurance) | Documented non-adherence | Intermittent (financial/insurance) | Documented non-adherence |
| **Severe exacerbations, prior year** | 1 | Not available | 3 | 3 | 2 | 0 |
| **Prior ICU admissions** | None documented | Multiple (3 in last year) | None documented | Multiple (pediatric) | Unknown | None documented |
| **Prior intubations** | None | 3 documented | None | None | Unknown | None |
| **Baseline spirometry (FEV1)** | FEV1 0.44 L (2011) | Pre/post-BD 40%/38% (2024) | Not available | Not available | Not available | Pre/post-BD 81%/84% (2016) |
| **Smoking / vaping history** | Daily THC, occasional nicotine, cocaine | 40 pack-year | Former, 3.75 pack-year | Denies | Cigar use (~2015) | Active vaping (recent) |
| **Blood eosinophils, cells/µL** | 162 | 74 | 87 | 141 | 39 | 96 |
| **Total IgE** | 5421 (2012) | Not available | Not available | Not available | Not available | Not available |
| **FeNO / atopy / nasal polyps / type 2 comorbidity** | Not assessed | Not assessed | Not assessed | Not assessed | Not assessed | Not assessed |
| **Prior or current biologic therapy** | Omalizumab approved and prescribed 2016, never initiated (patient declined) | None | None | None | None | None |
| **Outpatient pulmonary follow-up** | Last visit 2017 | Followed, non-adherent to plan | No outpatient evaluation (inpatient consult only) | Lost to follow-up (last visit 2013, pediatric) | Referred 2015, lost to follow-up | Referred after discharge; controller escalated |

Baseline characteristics were abstracted retrospectively from the electronic medical record and from available outside-hospital records. Several variables were incompletely documented in the retrospective record. FeNO, formal atopic status, nasal polyp status, and other type 2 comorbidities were not assessed in any patient, and total IgE was available for one patient only; these are reported as not available. Adherence was characterized from documented prescription-refill and clinic notes. Blood eosinophil counts reflect the absolute count nearest to admission and were below 300 cells/µL in all patients. Across the cohort, adherence was intermittent or documented as non-adherent in all six patients, and no patient was receiving biologic therapy at presentation; one patient (Case 1) had been approved for and prescribed omalizumab in 2016 but never initiated it. Baseline spirometry values were abstracted from prior clinical documentation rather than from primary pulmonary function test reports, were not uniformly available, and in some cases predate the index admission by several years; they should therefore be interpreted as historical and approximate. Asthma phenotype or label reflects the clinical diagnosis recorded in prior documentation and is historical; it was not derived from a standardized phenotyping or endotyping workup during this admission.

Abbreviations: BD = bronchodilator; COPD = chronic obstructive pulmonary disease; ED = emergency department; FeNO = fractional exhaled nitric oxide; FEV1 = forced expiratory volume in 1 second; ICS = inhaled corticosteroid; ICU = intensive care unit; IgE = immunoglobulin E; LABA = long-acting beta-2 agonist; LAMA = long-acting muscarinic antagonist; MDI = metered-dose inhaler; OSH = outside hospital; PRN = as needed; SABA = short-acting beta-2 agonist; THC = tetrahydrocannabinol.
